# Update on *IJTLD OPEN*: the future is open

**DOI:** 10.5588/ijtldopen.24.0316

**Published:** 2024-07-01

**Authors:** H.D. Blackbourn, G.B. Migliori

**Affiliations:** ^1^ International Union Against Tuberculosis and Lung Disease, Paris, France;; ^2^ Istituti Clinici Scientifici Maugeri, Istituto di Ricovero e Cura a Carattere Scientifico, Tradate, Italy

**Keywords:** tuberculosis, lung health, open access, equity, IJTLD, PubMed Central, LMICs

## Abstract

The launch of *IJTLD OPEN*, which is fully compliant with Plan S, has extended our author base and allowed readers worldwide to access the content for free. PubMed Central (PMC) has recently approved the journal for indexing (including indexing by PubMed), which will further improve visibility and access. Because authors retain copyright they can use their articles without restriction (e.g., to post on free digital repositories), helping to further disseminate their research. All these factors help to ensure that *IJTLD OPEN* has maximum reach and impact. However, we recognise that fees for open access may present a barrier for authors based in low- to middle-income countries. We call on the international community to ensure funding support for open access is broadly available, with equal opportunity for researchers worldwide.

Six months on from the launch of IJTLD OPEN, we thought it timely to update you on the progress achieved. We had previously outlined our commitment to the quality of peer review and the publication processes of the new journal,^1,2^ and we are delighted to report that the community has quickly embraced open access (OA) publishing. The fact that *IJTLD OPEN* is fully compliant with Plan S has encouraged a broad range of authors to write for us.^3^ This is vitally important as the extended author base has allowed us to publish articles on lung health in Africa,^4,5^ South America,^6^ Asia^7^ and Eastern Europe.^8^ In this way, the content of *IJTLD OPEN* complements the coverage provided by the *IJTLD*, but with the advantage that readers in settings that lack subscription access to the IJTLD can read the content for free: an immediate benefit of OA. There is also increasing awareness of the positive role that the broader community can play in tackling TB,^9^ but subscription-based models are a barrier to engaging these readers. This is fully resolved for OA and all relevant articles can be read by patients, carers, policymakers and civil society.

Another notable milestone is the recent approval by PubMed Central (PMC) for the journal to be indexed, including indexing by PubMed. This will further improve visibility and access. And because authors retain copyright, they can use their articles without restrictions in teaching materials, or to post on institutional websites or sites such as ResearchGate. These free digital repositories allow authors to engage with other researchers, which helps knowledge dissemination and increases the number of citations for articles.^10,11^ Authors for *IJTLD OPEN* can therefore be confident that their article has maximum reach.

We are grateful to funding agencies, which have been highly supportive of the OA format – and two sponsored series will launch later this year. We are also in the process of commissioning a new series, ‘Regional Perspectives on TB control’ to highlight the challenges that need to be addressed. We have asked the authors to be transparent and candid about the difficulties they face and have been delighted by their positive response – the first articles will be published later this year. This will be followed by a second phase highlighting the measures that can be applied across similar countries or regions to improve TB control.

Our sincere thanks to our Editorial Board for their support – and to our authors for helping to establish this new OA resource for respiratory health. However, we recognise that OA publishing models (and the requirement for article-processing fees) may present a barrier for authors in low- to middle-income countries (LMICs). We have removed charges for colour figures and reduced the OA fee by 25% – and the Union continues to subsidise the IJTLD as a free-to-publish journal for authors primarily (but not exclusively) in LMICs. Nevertheless, there is a need for further support, particularly in settings with a high burden of TB. As others have done, we call on the international community to ensure there is broad funding support for OA fees,^12^ providing equal opportunity for researchers worldwide.
